# Health-related quality of life impact of a triple combination of olmesartan medoxomil, amlodipine besylate and hydrochlorotiazide in subjects with hypertension

**DOI:** 10.1186/s12955-015-0216-6

**Published:** 2015-02-21

**Authors:** Pedro Marques da Silva, Uwe Haag, Julian F Guest, John E Brazier, Marco Soro

**Affiliations:** Arterial Investigation Unit, Medicine 4, Santa Marta’s Hospital, CHLC, Lisbon, Portugal; HaaPACS GmbH, Schriesheim, Germany; Catalyst Health Economics Consultants, Northwood, UK; Faculty of Life Sciences and Medicine, King’s College, London, UK; Health Economics and Decision Science (HEDS), School of Health and Related Research (ScHARR), University of Sheffield, Sheffield, UK; Health Economics & Outcomes Research Department, Daiichi-Sankyo Europe, Munich, Germany

**Keywords:** Olmesartan, Adherence, HRQoL, Hypertension, Pill burden

## Abstract

**Background:**

A post-hoc analysis was performed on the data from a 54 weeks phase III study (ClinicalTrials.gov identifier: NCT00923091) to measure changes in the health-related quality of life (HRQoL) of 2,690 patients aged ≥18 with moderate-to-severe hypertension who received one of six doses of olmesartan/amlodipine/hydrochlorothiazide (OLM/AML/HCTZ), using the MINICHAL and EQ-5D instruments.

**Methods:**

Descriptive statistics were used to assess blood pressure and HRQoL scores over the study period. Analysis of covariance (ANCOVA) was used to identify those factors that could possibly have influenced HRQoL. Linear regression was used to assess the relationship between changes in blood pressure and HRQoL scores.

**Results:**

Patients’ baseline MINICHAL mood and somatic domains scores were 5.5 and 2.6. Over the study period HRQoL improved as both MINICHAL scores decreased by 31-33%. Patients’ baseline EQ-5D index and VAS scores were 0.9 and 73.4 respectively, increasing by 6% and 12% over the study period. Patients’ QALY gain over the 54 weeks study period was estimated to be 0.029 QALYs. The ANCOVA showed that changes in patients’ HRQoL was likely to have been influenced by patients’ achievement of blood pressure control, the amount of concomitant medication and patients’ last used dosage strength of antihypertensive. Linear regression showed that blood pressure improvement may have been associated with improved HRQoL.

**Conclusions:**

This study showed that OLM/AML/HCTZ reduced blood pressure and significantly increased blood pressure control whilst improving patients’ HRQoL. Achieving blood pressure control, amount of concomitant medication and dosage strength of antihypertensive impacted on patients’ HRQoL.

## Background

The risk of cardiovascular disease among hypertensive individuals has been well documented [1], and hypertension for many is an asymptomatic disease. Recent evidence suggests that hypertension may contribute to reduce patients’ health-related quality of life (HRQoL) when compared to that of normotensive individuals [2]. Moreover, comorbid diseases in those with arterial hypertension can lead to a worsening of HRQoL when compared with hypertensive patients without any comorbidities [2-5]. Some antihypertensive medications have now been shown to worsen the HRQoL of hypertensive patients [6]. However, patients receiving antihypertensive therapy who reach their target blood pressure have an improved HRQoL compared to untreated patients and patients who do not reach their goal [6].

It has been estimated that 15–20% of hypertensive patients are not adequately controlled on a dual antihypertensive combination and that three or more different antihypertensive drug classes are required to achieve blood pressure control [6-8]. The triple combination antihypertensive therapy olmesartan/amlodipine/hydrochlorothiazide (OLM/AML/HCTZ; Sevikar HCT) has been shown to provide significantly greater blood pressure control compared with each of the component dual combinations (i.e. OLM/AML, OLM/HCTZ and AML/HCTZ) [9].

In a phase III, multicentred, double-blind, parallel-group design study (ClinicalTrials.gov identifier: NCT00923091), 2,690 patients aged ≥18 years with moderate-to-severe hypertension were randomised to receive placebo, OLM/AML (20 mg/5 mg), OLM/AML (40 mg/5 mg) or OLM/AML (40 mg/10 mg) for a period of 2 weeks [10]. Patients were then allocated to one of eight groups for a further 8-week period by being randomised to continue with the same dose of OLM/AML, or have HCTZ (12.5 mg or 25 mg) added to their treatment.

The second stage of the study constituted an open-label extension to assess the long-term efficacy and safety of the triple combination. Of the 2,543 patients who completed the double-blind phase, 2,540 patients initially underwent 8 weeks of single-blind treatment with OLM/AML/HCTZ (20 mg/5 mg/12.5 mg), after which 2,509 were entered into an open-label treatment phase. Of these, 2,439 patients (97.2%) completed the study [11].

Patients who responded to single-blind treatment with OLM/AML/HCTZ (20 mg/5 mg/12.5 mg) and achieved their target blood pressure (defined as mean seated systolic blood pressure/diastolic pressure (SeSBP/DBP) <140/90 mmHg, or <130/80 mmHg for those with diabetes or chronic kidney or cardiovascular disease) at week 18 continued with open-label treatment of OLM/AML/HCTZ (20 mg/5 mg/12.5 mg) for 36 weeks [11].

Patients who failed to achieve an adequate response to OLM/AML/HCTZ (20 mg/5 mg/12.5 mg) at week 18 were entered into the first of two consecutive 4-week periods of double-blind treatment which began with re-randomisation (1:2 ratio) to continue with OLM/AML/HCTZ (20 mg/5 mg/12.5 mg) or up-titration to the 40 mg/5 mg/12.5 mg dose. At the end of this 4-week period patients whose blood pressure was uncontrolled on OLM/AML/HCTZ (20 mg/5 mg/12.5 mg) were up-titrated to the 40 mg/5 mg/12.5 mg dose, while those who were controlled continued with their existing treatment. At week 26, all patients who had received the two consecutive 4-week periods of double-blind treatment entered 28 weeks of open-label treatment with OLM/AML/HCTZ (40 mg/5 mg/25 mg). Following the start of the open-label treatment phase, patients had their OLM/AML/HCTZ doses titrated with an aim to achieve blood pressure control at the investigator’s discretion with either of the following dose combinations: 20 mg/5 mg/12.5 mg, 40 mg/5 mg/12.5 mg, 40 mg/5 mg/25 mg, 40 mg/10 mg/12.5 mg or 40 mg/10 mg/25 mg. Up- and down-titration was allowed anytime based on a patient’s response to their antihypertensive dose [11].

The 8 week double-blind phase study showed that patients in every triple OLM/AML/HCTZ group had significantly greater mean reductions in diastolic and systolic blood pressure compared with patients on the corresponding OLM/AML therapy dose. During open-label treatment with OLM/AML/HCTZ, mean SeSBP/SeDBP levels remained within the ranges 120–140 and 75–85 mmHg, respectively. At the end of the study, significant reductions from baseline were seen in each group for SeSBP (37–43 mmHg) and SeDBP (22–27 mmHg), and 78.1% of patients achieved their blood pressure goal. At baseline, 90.8% of patients had moderate or severe hypertension, but at the study end 91.9% had normal/high normal blood pressure. The incidence of adverse events was similar across all treatment groups [11].

The primary aim of this phase III study was to evaluate the efficacy, safety and tolerability of the dual and triple combination therapies, and this has been reported elsewhere [11]. A secondary aim was to measure patients’ HRQoL over the study period. In accordance with protocol, of the 2,690 patients who were randomised in the double-blind study [10], 2,679 patients participated in the HRQoL component of the study .

## Methods

### Health-related quality of life

A post-hoc analysis of the data collected during the phase III trial (ClinicalTrials.gov identifier: NCT00923091) enabled an estimation of the HRQoL of 2,690 patients aged ≥18 with moderate-to-severe hypertension who received one of six doses of olmesartan/amlodipine/hydrochlorothiazide (OLM/AML/HCTZ). Due to the exploratory nature of the analysis, and the fact that the statistical analysis plan was prepared after the clinical report had been completed, the study is subject to several limitations.

The protocol, amendments and informed consent documents were submitted to, and approved by, the Independent Ethics Committee for each centre, prior to study initiation. Written informed consent was received from all patients prior to admission into the study, and investigators ensured that the study was conducted in accordance with the Declaration of Helsinki [10]. Patient reported outcomes were recorded using two health-related quality of life instruments: EQ-5D [12] and MINICHAL [13].

The EQ-5D is a generic instrument that measures patients’ responses across 5 dimensions of health (mobility, self-care, usual activities, pain/discomfort, and anxiety/depression). Each dimension has 3 answers to choose from, which can be generally classified as no problems, moderate problems, or significant problems on the specific dimension [12]. A total of 243 possible health states can be defined in this way, each with a unique 5 digit code. By applying a formula that attaches weights to each of the levels in each dimension the 5 digit code is converted into a single preference-based health index on a scale from 0 (health state as bad as being dead) to 1 (full health defined by EQ-5D) [12]. The EQ-5D scores were estimated from the preferences of a large general population in the UK [14]. The EQ-5D also includes a visual analog scale which is used to describe a patient’s own health state with scores ranging from 0 (worst state) to 100 (best state). When completing the instrument, patients were asked to describe their own health state on the day the questionnaire was answered [12].

The MINICHAL instrument consists of 16 items and measures the impact of hypertension on a patient’s HRQoL. The first 10 items are generally grouped to describe a patient’s mental state (mood domain) and the last 6 items are designed to evaluate their somatic manifestations (somatic domain), i.e. the potential physical effects of hypertension. Each question has 4 response options: 0 = No, not at all; 1 = Yes, somewhat; 2 = Yes, quite a lot; and 3 = Yes, a great deal. The score for each dimension is obtained by summing the scores of the items within that dimension and scores range from 0 (best HRQoL) to 30 (worst HRQoL) for the mood domain and from 0 to 18 for the somatic domain [13].

For MINICHAL, patients’ completed scores were used to calculate their mood domain, somatic domain, and total scores according to the instrument’s specifications and guidelines in order to evaluate changes in patients’ HRQoL during the study [13]. For the EQ-5D, patients’ scores were converted to utilities according to the instrument’s specifications and guidelines [12]. Patients completed these two instruments on four occasions: baseline, week 10, week 26 and week 54. Changes in HRQoL from baseline to week 54 are reported as changes in the following target variables: EQ-5D index, EQ-5D visual analogue scale, MINICHAL mood domain, MINICHAL somatic domain and MINICHAL total score.

### Statistical analyses

The analyses were conducted in sequential steps.

The first step involved the generation of descriptive statistics for the following quantitative factors:

- Age at baseline (≤60 years / elderly >60 years);
- Gender;
- Weight based on BMI at baseline (normal [≤25 kg/m^2^], over-weight [>25 kg/m^2^ to ≤30 kg/m^2^] and obese [>30 kg/m^2^]);
- Change in systolic blood pressure in quartiles from baseline to Week 54;
- Change in diastolic blood pressure in quartiles from baseline to Week 54;
- Secondary prevention for cardiovascular risk factors (diabetes, cardio-vascular disease, renal impairment);
- Smoking status (Non-Smoker/Ex-Smoker/Current Smoker);
- Country (Eastern EU/Central-Western EU);
- Concomitant cardiovascular risk using concomitant medications as a proxy;
- Mental disorder using concomitant medications as a proxy;
- Number of concomitant medications per patient during the study;
- Baseline hypertension grade (Grade 1 hypertension SBP 140–159 mmHg or DBP 90–99 mmHg; Grade 2 hypertension SBP 160–179 mmHg or DBP 100–109 mmHg; Grade 3 hypertension SBP ≥180 mmHg or DBP ≥110 mmHg);
- Blood pressure goal control at week 54 (SBP/DBP ≤130/80 mmHg for diabetic patients, renally impaired patients, or patients with cardiovascular disease; SBP ≤140/90 mmHg for all other patients).

The second step involved performing analysis of covariance (ANCOVA) for each target variable and factor, to assess if these factors possibly influenced HRQoL changes during the study. Factors with p-value of >0.2 were omitted in the third step. Any factors with a p-value for effect of 0.2 or lower were used in the third step for the respective target covariate. The third step involved stepwise ANCOVA that included treatment and all pre-selected factors for each target variable based on second step findings until all remaining factors had p-values ≤0.05.

The outputs of the analyses were a descriptive summary of the results. P-values between 0.05 and 0.01 were considered a trend and p-values of 0.01 or lower were considered to be indicative. Since multiple tests were performed without any adjustment, the p-values even if statistically significant, should only be considered to be indicative of trends or possible hypotheses.

Since blood pressure changes from baseline to week 54 were initially not included in the ANCOVA models, a separate linear regression model was calculated to assess the relationship between changes in blood pressure and HRQoL scores from baseline.

## Results

Of the 2,690 patients who were randomised into the double-blind study [10], 2,679 patients participated in the HRQoL component of the study. The demographic and baseline characteristics of the 2,679 patients who completed the HRQoL instruments at baseline are shown in Table 1. The mean age of patients was 56.2 years; mean weight was 88.5 kg; 53.7% were female [11]; 14.6% were diabetic; and 28.70% had cardiovascular disease (Table 2). The mean baseline SeSBP/SeDBP was 168.4/103.8 mmHg and 89.7% of patients had SBP between 140 and 179 mmHg or DBP between 90 and 109 mmHg (Grade 1 or 2 hypertension) whilst 10.3% had SBP of 180 or DBP of 110 mmHg (Grade 3 hypertension) and the mean duration of disease (hypertension) was 8.3 years.

**Table 1:** **Patients’ characteristics**

	**Number of patients (percentage of sample in parentheses)**
Age	
<=60 years	1743 (65.1%)
>60 years	936 (34.9%)
Gender	
Female	1439 (53.7%)
Male	1240 (46.3%)
Body mass index	
<=25 kg/m2	309 (11.5%)
>25 to < =30 kg/m2	1003 (37.4%)
= > 30 kg/m2	1367 (51.0%)
Smoking status	
Current smoker	464 (17.3%)
Ex-smoker	301 (11.2%)
Non-smoker	1914 (71.4%)
Country	
Eastern European countries	1763 (65.8%)
Western-central European countries	916 (34.2%)

**Table 2:** **Patients’ clinical profile**

	**Number of patients (percentage of sample in parentheses)**
Baseline hypertension	
Grade 1	27 (1.0%)
Grade 2	2375 (88.7%)
Grade 3	277 (10.3%)
Diabetes	
No	2287 (85.4%)
Yes	392 (14.6%)
Cardiovascular disease	
No	1911 (71.3%)
Yes	768 (28.7%)
Renal impairment	
No	2625 (97.9%)
Yes	54 (2.0%)
Cardiovascular risk	
Yes	566 (21.1%)
No	2113 (78.9%)
Mental Disorder	
Yes	153 (5.7%)
No	2526 (94.3%)
Concomitant medications	
0	417 (15.6%)
1-2	857 (32.0%)
3-4	635 (23.7%)
5-6	371 (13.9%)
= > 7	399 (14.9%)
Achieved hypertension goal by week 54	
Missing data	171 (6.4%)
No	549 (20.5%)
Yes	1959 (73.1%)

The efficacy and safety of the alternative antihypertensive combinations have been described elsewhere [10,11]. However, it is noteworthy that differences were observed in the baseline characteristics of patients who ended the study in different treatment groups. For example, the low dose OLM/AML/HCTZ 20/5/12.5 mg group contained the lowest proportion of males, current smokers, diabetics and patients with cardiovascular disease. In contrast, the higher dose OLM/AML/HCTZ 40/10/12.5 and 40/10/25 mg groups contained more males and higher proportions of diabetics and patients with cardiovascular disease.

**Figure 1:**
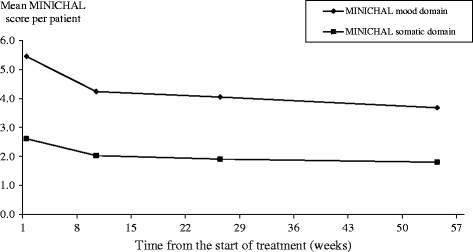
**Mean MINICHAL scores per patient at different times from the start of antihypertensive treatment.** Not all patients completed all HRQoL instruments at each time point.

**Figure 2:**
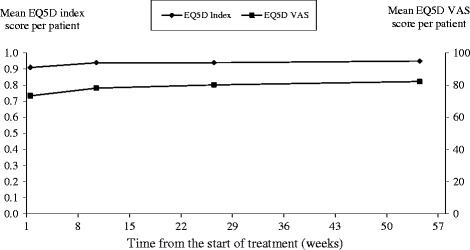
**Mean EQ-5D scores per patient at different times from the start of antihypertensive treatment.** Not all patients completed all HRQoL instruments at each time point.

At baseline patients’ MINICHAL mood domain and somatic domain scores were 5.5 and 2.6 respectively. Over the 54 weeks study period patients’ HRQoL improved as both MINICHAL scores decreased by 31-33% (Figure 1). Patients’ baseline EQ-5D index and VAS score was 0.9 and 73.4 respectively, (Figure 2) increasing by 6% and 12% respectively over the study period (Table 3). The mean difference in the EQ-5D index score from baseline to week 54 was assessed with a univariate procedure to calculate the change in quality-adjusted life year (QALY). Patients’ QALY gain over the 54 weeks study period was estimated to be 0.029 QALYs.

**Table 3:** **Patients’ mean (standard deviation) blood pressure and health-related quality of life status** ^*^

	**Baseline**	**Week 54**	**Difference between week 54 and baseline**	**p value derived using a t-test**
Systolic blood pressure	168.4 (6.6)	127.1 (9.0)	−41.3 (10.0)	<0.0001
Diastolic blood pressure	103.8 (2.9)	78.0 (6.6)	−25.9 (6.9)	<0.0001
EQ-5D index	0.90 (0.13)	0.95 (0.11)	0.05 (0.12)	<0.0001
EQ-5D VAS	73.4 (15.0)	82.3 (12.5)	8.9 (14.4)	<0.0001
MINICHAL mood domain	5.45 (4.42)	3.68 (3.74)	−1.80 (4.24)	<0.0001
MINICHAL somatic domain	2.62 (2.66)	1.80 (2.16)	−0.80 (2.46)	<0.0001
MINICHAL total score	8.00 (6.22)	5.40 (5.12)	−2.60 (5.68)	<0.0001

^*^Not all patients completed all HRQoL instruments at each time point.

**Figure 3:**
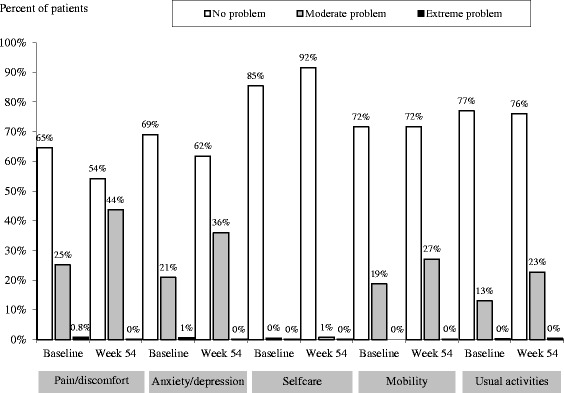
**Percentages of patients reporting problems across each EQ-5D index dimension at baseline and after 54 weeks of treatment.** Data was missing from 10% of patients at baseline and 1% of patients at week 54.

The majority of patients reported no problems in the different EQ-5D dimensions, although more patients reported experiencing pain/discomfort and anxiety/depression than problems with the other dimensions (Figure 3).

Results from Step 2 of the ANCOVA showed that renal impairment and smoking status were unlikely to have influenced the HRQoL changes observed during the study, since their p value was >0.2. Hence, they were omitted from the ANCOVA model in the third step. Blood pressure changes in quartiles were also excluded from the third step as that was pre-determined by the analysis protocol. The factors/endpoints with a p value of <0.05 after performing Step 3 of the ANCOVA are summarised in Tables 4 and 5. Whilst these p values are explorative only, they are indicative of those baseline factors that influenced the patients’ HRQoL status at the end of the study period. Nevertheless, there were very slight differences in the change in patients’ HRQoL over the study period when stratified by the different baseline factors. For example, the change in HRQoL was similar irrespective of patients’ age, gender, BMI, etc. The one exception to this was in patients’ baseline hypertension grade. The change in HRQoL for patients with Grade 2 and 3 hypertension was similar, but different to that of patients with Grade 1 when assessed using the EQ-5D VAS. However, patients’ baseline hypertension grade was found not to have influenced HRQoL when assessed using the MINICHAL. Similarly, neither the presence of cardiovascular disease nor patients’ use of concomitant medication for cardiovascular disease or mental disorder appeared to influence their HRQoL when assessed by MINICHAL. Conversely, the amount of concomitant medication (“pill burden”), blood pressure target achievement and patients’ antihypertensive treatment in period 6 influenced HRQoL when assessed by the EQ-5D but not when assessed by the MINICHAL. Notwithstanding this, patients’ baseline HRQoL score derived from each scale influenced their HRQoL score on that same scale at week 54.

**Table 4:** **Results of Step 3 from the ANCOVA investigating the relationship between sociodemographic factors and morbidity on patients’ health-related quality of life measured using the EQ-5D**

**Endpoint/Factors**	**EQ-5D Index**			**EQ-5D VAS**		
	**Baseline**	**Difference between week 54 and baseline**	**p value**	**Baseline**	**Difference between week 54 and baseline**	**p value**
Age class			<0.0001			
<=60 years	0.92 (0.12)	0.00 (0.12)				
>60 years	0.89 (0.14)	0.00 (0.13)				
Gender			0.0005			0.0002
Male	0.94 (0.11)	0.00 (0.10)		75.6 (14.2)	−8.50 (13.59)	
Female	0.89 (0.15)	0.00 (0.14)		71.4 (15.4)	−9.30 (15.08)	
BMI			0.0426			
<=25 kg/m2	0.94 (0.11)	0.00 (0.12)				
>25 to < =30 kg/m2	0.92 (0.13)	0.00 (0.12)				
= > 30 kg/m2	0.91 (0.13)	0.00 (0.13)				
Diabetes						0.0101
Yes				69.2 (15.4)	−8.60 (15.91)	
No				74.1 (14.8)	−8.90 (14.14)	
Cardiovascular disease						<0.0001
Yes				70.4 (14.5)	−10.90 (13.83)	
No				74.5 (15.5)	−8.10 (14.55)	
Country (region)						0.0085
Western-central countries				76.0 (15.5)	−7.80 (15.50)	
Eastern countries				72.0 (14.5)	−9.40 (13.81)	
Concomitant medication for cardiovascular disease			0.0065			
Yes	0.91 (0.13)	0.00 (0.12)				
No	0.92 (0.12)	0.00 (0.13)				
Concomitant medication for mental disorder			0.021			0.0263
Yes	0.88 (0.17)	0.00 (0.19)		68.7 (17.8)	−10.40 (18.05)	
No	0.92 (0.13)	0.00 (0.12)		73.6 (14.7)	−8.80 (14.22)	
Hypertension grade at baseline						0.0258
Grade 1				80.6 (11.8)	−1.10 (11.09)	
Grade 2				73.4 (15.0)	−8.80 (14.25)	
Grade 3				72.4 (15.2)	−10.40 (15.67)	
Achieved hypertension control by week 54			0.0019			
Yes	0.92 (0.13)	0.00 (0.12)				
No	0.89 (0.15)	0.00 (0.14)				
Baseline value of respective endpoint			<0.0001			<0.0001
SeSBP changes from baseline to week 54			0.0010			0.0160
SeDPB changes from baseline to week 54			0.0039			0.0120

*The baseline values (standard deviation) and differences (standard deviation) between week 54 and baseline were calculated independently of the Step 3 model.

**Table 5:** Results of Step 3 from the ANCOVA investigating the relationship between sociodemographic factors and morbidity on patients’ health-related quality of life measured using the MINICHAL*

**Endpoint/Factors**	**MINICHAL mood domain**			**MINICHAL somatic domain**			**MINICHAL total score**		
	**Baseline**	**Difference between week 54 and baseline**	**p value**	**Baseline**	**Difference between week 54 and baseline**	**p value**	**Baseline**	**Difference between week 54 and baseline**	**p value**
Age class			<0.0001			0.0058			<0.0001
<=60 years	5.34 (4.38)	−2.00 (4.21)		2.51 (2.68)	−0.80 (2.46)		7.80 (6.24)	−2.80 (5.67)	
>60 years	5.63 (4.48)	−1.40 (4.27)		2.81 (2.61)	−0.70 (2.45)		8.38 (6.16)	−2.00 (5.66)	
Gender			0.0151			0.0004			0.0045
Male	4.74 (4.10)	−1.60 (4.00)		2.18 (2.34)	−0.70 (2.22)		6.88 (5.66)	−2.30 (5.38)	
Female	6.06 (4.59)	−1.90 (4.43)		2.99 (2.86)	−0.90 (2.64)		8.99 (6.51)	−2.80 (5.92)	
BMI						0.0063			
<=25 kg/m2				2.32 (2.50)	−0.80 (2.03)				
>25 to < =30 kg/m2				2.33 (2.53)	−0.80 (2.40)				
= > 30 kg/m2				2.89 (2.76)	−0.80 (2.58)				
Diabetes			0.0326						
Yes	5.80 (4.70)	−1.40 (4.52)							
No	5.38 (4.37)	−1.90 (4.19)							
Country (region)			<0.0001						<0.0001
Western-central countries	4.56 (4.66)	−1.80 (4.42)					6.70 (6.45)	−2.30 (5.97)	
Eastern countries	5.90 (4.22)	−1.80 (4.14)					8.68 (5.98)	−2.70 (5.52)	
Amount of concomitant medication			0.0387			<0.0001			0.0002
0	5.79 (4.27)	−2.20 (4.30)		2.43 (2.42)	−1.00 (2.05)		8.21 (5.77)	−3.20 (5.24)	
1-2	5.44 (4.32)	−1.90 (3.92)		2.32 (2.55)	−0.80 (2.38)		7.69 (6.00)	−2.70 (5.25)	
3-4	5.15 (4.22)	−1.80 (4.02)		2.73 (2.78)	−0.80 (2.53)		6.00 (5.06)	−2.50 (5.70)	
5-6	5.38 (4.37)	−1.30 (4.40)		2.91 (2.78)	−0.70 (2.55)		8.28 (6.22)	−2.00 (5.97)	
= > 7	5.61 (5.08)	−1.50 (5.01)		3.01 (2.75)	−0.60 (2.85)		8.57 (7.00)	−2.00 (6.77)	
Achieved hypertension control by week 54						0.002			0.011
Yes				2.44 (2.59)	−0.80 (2.37)		7.75 (6.13)	−2.70 (5.62)	
No				3.13 (2.85)	−0.70 (2.77)		8.67 (6.33)	−2.20 (5.90)	
Treatment in Period 6			0.0158			0.0012			0.024
OM20/AML5/HCTZ12.5	5.19 (4.24)	−1.90 (4.23)		2.39 (2.59)	−0.80 (2.32)		7.48 (5.99)	−2.60 (5.59)	
OM40/AML5/HCTZ12.5	5.94 (5.05)	−1.40 (4.80)		3.02 (3.04)	−1.00 (3.02)		8.93 (7.09)	−2.40 (6.81)	
OM40/AML5/HCTZ25	5.94 (4.58)	−2.00 (4.12)		2.67 (2.58)	−0.90 (2.49)		8.59 (6.22)	−2.90 (5.50)	
OM40/AML10/HCTZ12.5	5.69 (4.39)	−1.70 (3.90)		3.19 (2.95)	−0.50 (2.77)		8.95 (6.46)	−2.30 (5.88)	
OM40/AML10/HCTZ25	4.83 (3.89)	−1.20 (3.88)		2.86 (2.43)	−0.50 (2.25)		7.67 (5.41)	−2.00 (4.73)	
Baseline value of respective endpoint			<0.0001			<0.0001			<0.0001
SeSBP changes from baseline to week 54			0.0089			0.0002			0.0006
SeDPB changes from baseline to week 54			0.1089			0.0080			0.0273

*The baseline values (standard deviation) and differences (standard deviation) between week 54 and baseline were calculated independently of the Step 3 model.

The factors included in the EQ-5D VAS step 3 model are not exhaustive and do not account for all of the observed difference in VAS score. The R^2^ for the ANCOVA model was 0.188. The different factors incorporated in the separate EQ-5D index, MINICHAL mood and MINICHAL somatic step 3 models are likely to be more influential over the changes in patients’ HRQoL and the R^2^ was 0.408, 0.431 and 0.448 respectively. Notwithstanding this, changes in the patients’ HRQoL was possibly influenced by other factors which were not measured in this study. Additionally, a separate linear regression analysis showed that blood pressure improvement may have been associated with improved HRQoL.

## Discussion

This study assessed the HRQoL of hypertensive patients who received one of six doses of OLM/AML/HCTZ to reduce their high blood pressure using both the EQ-5D and MINICHAL instruments over 54 weeks including the open label phase. At the start of the study, 90.8% of patients had Grade 2 or 3 hypertension, but at the study end 91.9% had achieved the SeSBP <140 mmHg threshold [11]. Furthermore, the incidence of adverse events was similar across the treatment groups [11]. Patients’ hypertension grade at baseline did not appear to affect their HRQoL, except the EQ-5D VAS score of those with Grade 3 disease was lower than that of the other patients. This may reflect the admission criteria of the clinical trial [10,11] since patients’ baseline health status was relatively high. In contrast, others have reported that patients with Grade 1 disease have better MINICHAL scores than those with Grade 2/3 [15,16].

Previous studies using the MINICHAL have shown that patients treated with antihypertensives who experienced reduced blood pressure experienced a greater decrease in MINICHAL scores than those patients who reported no overall change in blood pressure. In this study, patients’ HRQoL improved since their MINICHAL scores decreased by 31-33% over the 54 weeks study period. However, what constitutes clinically relevant changes in MINICHAL scores is currently undecided. The mean mood and somatic MINICHAL scores among a normotensive population in Brazil have been estimated at 3.2 and 0.8 respectively [17]. The corresponding values among the hypertensive population in the Brazilian study was 5.3 and 1.9 respectively [17]. Other studies that have used the MINICHAL reported mean mood scores of 6.6 [18] and 5.6 [15] and mean somatic scores of 5.0 [18] and 2.1 [15]. In the present study the mean mood and somatic baseline scores of 5.5 and 2.6 respectively were concordant with those from the other studies [15,17,18]. Notwithstanding the variance between studies, use of the MINICHAL detected that the patients in the present study had a poorer HRQoL than would be expected from normotensive patients, which is consistent with the finding of others [19]. Moreover, the somatic domain appeared to be more sensitive than the mood domain in the detecting changes in patients’ HRQoL in this study.

The ANCOVA model showed that multiple factors influenced HRQoL changes. However, individual categorical variables (as identified in step 3) would seem to have only accounted for up to 40% of the variation in patients’ HRQoL (measured using the MINICHAL), since R^2^ was of the order of 0.4. This value indicates that other variables (such as socioeconomic factors and stress which were not measured in the clinical trials) have not been included in the ANCOVA model and thus it was not possible to control for their effect. Nevertheless, achievement of blood pressure target was found to be an influencer for HRQoL. Both these variables have been shown to be independent predictors of HRQoL among hypertensive patients [20,21]. Also, step 3 of the MINICHAL model suggests that the amount of co-medication (“pill burden”) affects patients’ HRQoL. Moreover, a high pill burden among hypertensive patients has been shown to adversely affect adherence [22,23]. Therefore, the findings from this study support the notion that physicians should prescribe in a manner to reduce their patients’ pill burden. Additionally, the HRQoL of patients treated with an anti-hypertensive in an observational study was found to significantly influence adherence to treatment [24]. Furthermore, patients with high adherence to their treatment schedule have a much higher chance of attaining their target blood pressure than patients with low adherence [25]. Consequently, adherence plays an important role in reducing the risk of developing cardiovascular events [26]. Moreover, achieving blood pressure control influenced patients’ HRQoL, as demonstrated by the step 3 EQ-5D index and MINICHAL somatic domain models, despite patients’ high baseline health status.

The mean EQ-5D index and VAS baseline scores for the whole study cohort was 0.9 and 73.4 respectively. These baseline scores are high, probably due to the fact that patients with major concomitant cardiovascular risk factors and co-morbidities were excluded by the clinical trial protocol. Nonetheless, these scores increased by 6% and 12% respectively over the study period. In comparison, the international population norm for the EQ-5D index across the 18 to 75+ age group has been reported to range from 0.74 to 0.95 and that of the EQ-5D VAS has been shown to range from 71.1 to 83.7 [27]. These comparisons suggest that the EQ-5D index score of this patient population is higher relative to population norms than the EQ-5D VAS, which may suggest that the EQ-5D index is not a sufficiently sensitive instrument with which to measure HRQoL among hypertensive patients with no major additional cardiovascular risk factors.

Notwithstanding this, the analysis showed that age, gender, BMI, whether patients were taking cconcomitant medication for cardiovascular disease and/or mental disorder and whether they achieved hypertension control by week 54 all influenced the EQ-5D index score. Additionally, gender, co-morbid diabetes, co-morbid cardiovascular disease, country of origin, whether patients were taking concomitant medication for mental disorder and their hypertension grade at baseline influenced their EQ-5D VAS score. The effect of concomitant cardiovascular diseases on HRQoL has been much debated in the published literature. For example, the epidemiological study ESTHER showed that in a large cohort of hypertensive patients being managed by general practitioners, additional cardiovascular risk factors did not seem to be related to a substantial worsening of their HRQoL measured using the SF-12. Nonetheless, the physical component score (PCS) reported in the ESTHER study was found to be significantly decreased by cardiovascular risk factors such as BMI, macroalbuminuria, diabetes and circulatory disorders [28]. In our phase III study study on OLM/AML/HCTZ the analysis of patients’ comorbidities suggested that the study population comprised a relatively healthy cohort with relatively few experiencing comorbid illness, reflecting the exclusion criteria of the trial. The possibility of a “ceiling effect” in EQ-5D index because of the high baseline values (37.3% of patients had an EQ-5D value of 1.00) leading to artifactual parameter estimates in the ANCOVA cannot be excluded. It was not considered appropriate to reduce the potential impact of a ceiling effect as available econometric methods [29] have been applied to simple ordinary least squares regression models, but not to ANCOVA models that incorporate covariates. Notwithstanding the limitations of using the EQ-5D instrument to detect HRQoL changes among hypertensive patients, it did detect important differences in those who had controlled their hypotension.

The Step 3 analyses identified multiple factors that were likely to have influenced HRQoL simultaneously. Blood pressure control was the only blood pressure-related factor modelled in Step 3. However, linear regression showed that blood pressure improvement may have been associated with improved HRQoL.

This post-hoc analysis is subject to several other limitations. The statistical analysis protocol for the post-hoc HRQoL analysis was prepared after the clinical report had been completed. Multiple statistical tests were performed without any adjustment. Hence, the p-values can only be viewed as being exploratory and indicative of trends or possible hypotheses. Furthermore, the large sample size may have resulted in small, statistically significant p values for changes that may have marginal clinical significance. Factors that may have influenced patients’ HRQoL, such as educational level, marital status, job status, income and residency, were not collected during the phase III trial. Exclusion of these factors should be considered as an additional limitation. Moreover, the large sample size of this non-comparative study has only generated small effects. Hence, the reproducibility of the HRQoL impact of OLM/AML/HCTZ seen in this study needs to be studied further in a controlled study design.

In conclusion, this present study showed that OLM/AML/HCTZ reduced blood pressure and significantly increased blood pressure control whilst improving patients’ HRQoL. Achieving blood pressure control, amount of concomitant medication and dosage strength of antihypertensive all impacted on patients’ HRQoL. Hence, when prescribing antihypertensive agents physicians should consider the impact of all these factors on patients’ HRQoL and on their adherence to treatment.
